# Early Forays Into Using Spinal Anesthesia for Inguinal Hernia Surgery in Preterm Infants: A Retrospective Analysis From a Single Center in Karnataka

**DOI:** 10.1002/pdi3.70015

**Published:** 2025-07-24

**Authors:** Ranganatha A. Devaranavadagi, Gayatri Sasikumar, Pavitra Gangadharan Chandrasekaran, Netra S. Kannur, H. A. Venkatesh, Suma Sriramanan, C. N. Radhakrishnan, Jayashree S. Simha, Karthik Nagesh

**Affiliations:** ^1^ Department of Neonatology, Department of Anaesthesia, Department of Pediatric Surgery Manipal Hospital Bangalore India

**Keywords:** bronchopulmonary dysplasia, general anesthesia, inguinal hernia, neonate, preterm, spinal anesthesia

## Abstract

Neonates, particularly those born preterm, have a higher incidence of inguinal hernia and are at increased risk of cardiopulmonary complications associated with general anesthesia. To mitigate these risks, spinal anesthesia has been adopted in our institution for over two decades as the preferred technique for inguinal hernia repair in neonates. This retrospective study presents a 6‐year experience with neonatal spinal anesthesia and includes both term and preterm infants undergoing inguinal surgery. We specifically analyzed cases in which spinal anesthesia was used in neonates with a postconceptional age below 50 weeks. Nineteen patients underwent inguinal surgery under spinal anesthesia in this timeframe. The gestational age at birth ranged from 27 to 38 weeks. The postmenstrual age at the time of surgery ranged from 35 to 46 weeks. The birth weight ranged from 740 to 3300 g, whereas the weight at surgery ranged from 1550 to 4900 g. A 26G hypodermic needle was used to give the spinal block, and 0.2 mL/kg of 0.5% heavy bupivacaine was injected. No cases required respiratory support and/or inotropic support during the procedure and postoperatively. None had apnea during/after surgery, including the four cases of bronchopulmonary dysplasia. None required general anesthesia. Spinal anesthesia for inguinal hernia repair is a safe and efficient method that obviates the necessity for NICU admission or any escalation in respiratory care, including in the cases of bronchopulmonary dysplasia.

## Graphical abstract

Preterm infants are at high risk of cardiopulmonary complications with general anesthesia during inguinal hernia repair. This study evaluated 19 preterm infants who underwent spinal anesthesia and found that none required respiratory or inotropic support. Spinal anesthesia was safe and effective, avoiding both NICU admission and respiratory escalation—even in infants with bronchopulmonary dysplasia.

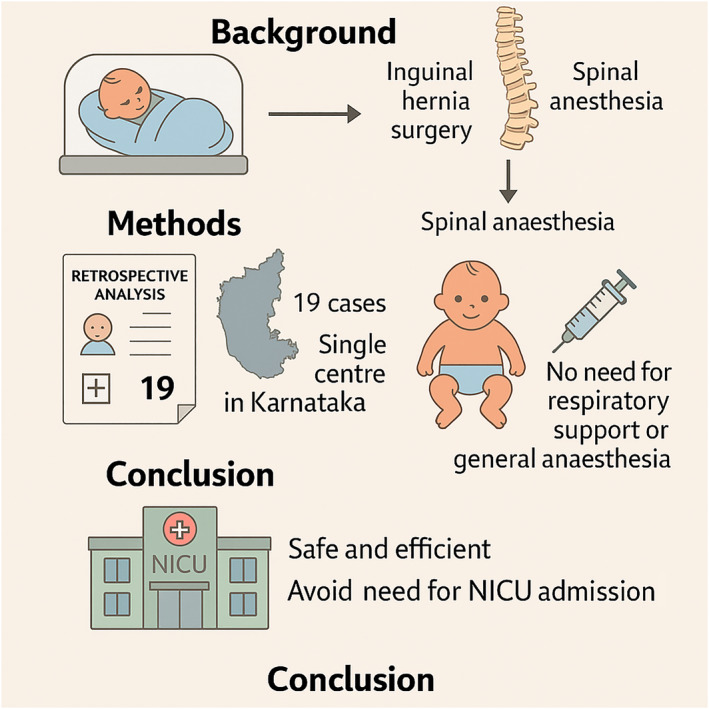

## 1 Introduction

With improvements in perinatal medicine and advanced neonatal intensive care, there is a substantial increase in preterm survival rate. In 2020, an estimated 13.4 million babies were born preterm (before 37 completed weeks of gestation) worldwide [1]. Surgery is necessary for a considerable proportion of former preterm babies. Specifically, 10%–30% of infants born preterm develop inguinal hernias [2]. Postoperative apnea, bradycardia, and desaturation are the common concerns among preterm infants undergoing inguinal surgeries, with general anesthesia (GA) requiring sometimes postoperative mechanical ventilation and the need for hospital stay [3, 4, 5, 6, 7].

In a study conducted by Frumiento et al., spinal anesthesia (SA) was found to be a safe and effective method for preterm infants in an outpatient setting, in contrast to general anesthesia, which results in postoperative apnea up to 30%. This approach not only eliminates the need for hospital admission but also reduces the costs associated with their care [8]. There is a paucity of data from India on the use of SA for inguinal hernia repair. This analysis aims to highlight our 6‐year experience in effectively utilizing SA in preterm infants, especially those suffering from bronchopulmonary dysplasia (BPD), to showcase its safety.

## 2 Methods and Clinical Procedure

Our tertiary caring neonatal referral unit has been managing extremely preterm infants for the past three decades. The unit is supported by a specialized pediatric anesthesia department, which possesses significant expertise in administering neonatal spinal anesthesia.

**FIGURE 1:**
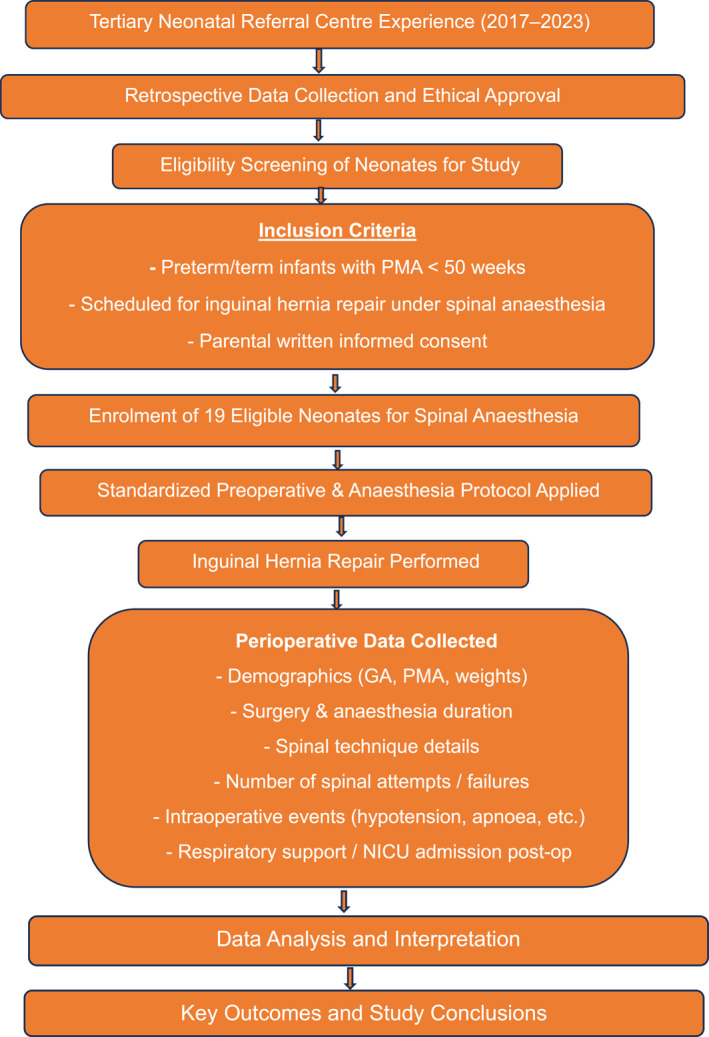
Flow chart of the study. GA, general anesthesia; NICU, neonatal intensive care unit; PMA, postmenstrual age.

This is a retrospective study involving data collection; the detailed exclusion criteria are in 2.1, and the inclusion criteria are depicted in Figure 1.

### 2.1 Exclusion Criteria

1. Infants in whom GA was used.
2. Presence of major congenital anomalies or neurological conditions affecting the feasibility of SA.
3. Preoperative coagulopathy or thrombocytopenia, making SA unsafe.
4. Neonates with known spinal abnormalities (e.g., myelomeningocele and sacral agenesis).
5. Cases where additional surgical procedures were performed beyond inguinal hernia repair.
6. Incomplete or missing perioperative data prevent analysis.

### 2.2 Clinical Subject Enrollment

Detailed information regarding the risks and methods of GA *versus* SA had been thoroughly communicated to the parents of these infants during the anesthesia consultation. The parents were requested to provide written consent for both the anesthesia procedure and the surgical intervention. A consensus was reached in 2002 to establish the subsequent anesthesia protocol, which has been consistently adhered to all of these patients up until the present time.

### 2.3 Definition of BPD Applied

National Institutes of Health (NIH) 2001: FiO_2_ requirement more than 21% for more than 28 days (used for the infants born in 2017).

NIH 2018: Infant born less than 32 weeks, with persistent lung parenchymal changes in X‐ray requiring respiratory support for 3 days or more at 36 weeks to maintain saturation of 90%–95% (used in infants born between 2018 and 2023).

### 2.4 Anesthesia Procedure

Routine preoperative evaluation, including cardiac and neurological assessment, complete blood count, and coagulation profile, was carried out as per the institute protocol. On the day of surgery, babies were kept *Nil Per Os* for 6 h for formula feeds and 4 h for breast milk. They were admitted to the neonatal intensive care unit (NICU) where intravenous access was secured, and intravenous fluid was started at a maintenance rate. One hour before the procedure, an eutectic mixture of lignocaine and prilocaine (EMLA) cream was applied on the lower back of the baby at the proposed site of lumbar puncture by the anesthesia team. In the operating room, care was taken to keep the baby warm, American Society of Anesthesiologists (ASA) standard monitors were connected, and a dextrose‐soaked pacifier was used to keep the infant quiet. Intravenous fluid was continued at the maintenance rate. If the baby was on oxygen or nasal continuous positive airway pressure (CPAP), the same procedure was continued intraoperatively. Lumbar subarachnoid block (SAB) was performed with the baby held in the left lateral position by a trained anesthesia technician who took special care to avoid airway obstruction. The EMLA patch was removed, and the area was prepped with warm betadine solution. A 26G hypodermic needle was used to give the block, and 0.2 mL/kg of 0.5% heavy bupivacaine was injected intrathecally using an insulin syringe. Time of administration of the spinal drug was noted, and the noninvasive blood pressure (NIBP) cuff was reapplied on the lower limb, taking care not to raise the legs to avoid spinal ascent. The babies were monitored throughout the procedure. No sedatives were given, and postoperatively they were shifted back to the NICU for observation for 2–4 h and discharged subsequently if no complications.

### 2.5 Analyzed Parameters and Variables

The study analyzed various parameters and variables, such as birth weight and birth gestation. Additionally, the study also considered other factors such as postmenstrual age (PMA), weight before surgery, the use of preanesthesia sedatives, the anesthesia duration/surgery duration, the frequency of failed SA attempts, and the occurrence of perioperative complications. These complications encompassed hypotension, the need for escalation of respiratory support, and the need for admission to the NICU. All of these variables were meticulously recorded for each case in the study. For the study, infants requiring NICU stay due to complications arising from anesthesia were considered NICU admissions. Figure 1 depicts a flowchart of the study.

The institutional ethical committee has issued no‐objection certificate (EC/RENEW/INST/2024/17014). No patient identification details have been revealed in the study. Informed consent for surgery/anesthesia was signed by parents in all cases.

## 3 Results

A total of 24 neonates were initially identified as candidates for inguinal hernia repair between 2017 and 2023. Of these:

Three neonates were excluded due to the use of GA (not meeting the inclusion criterion). One neonate had associated neurological anomalies precluding SA (exclusion criterion). One case had incomplete perioperative documentation (exclusion criterion).

### 3.1 Baseline Parameters

Out of 19 cases, 2 cases were in NICU (Cases 3 and 12: preterm care with BPD both on heated humidified high flow nasal cannula support), and the remaining cases were admitted for surgery. The gestational age at birth ranged from 27 to 38 weeks, with a mean of 31.94 ± 3.55 weeks. The PMA at the time of surgery ranged from 35 to 46 weeks, with a median (IQR) of 39 (± 6.5) weeks. The birth weight ranged from 0.74 to 3.30 kg, with a mean of 1.56 ± 0.75 kg, whereas the weight at surgery ranged from 1.55 to 4.90 kg, with a mean of 2.87 ± 0.98 kg. The SA administration was successful for all cases.

### 3.2 Clinical Procedure

The range of surgical duration was 25–52 min, with a median of 34 ± 8 min. The duration of SA lasted for the complete procedure in all cases. Blood pressure remained stable throughout the procedure, with no instances of hypotension requiring intervention. Mean arterial pressure (MAP) remained within the normal range for the corresponding gestational age from preoperative to postoperative periods. No episodes of intraoperative or postoperative apnea were noted. The other details are mentioned in Table 1.

**TABLE 1:** Baseline clinical characteristics of study population.

	Mean ± SD	Median (IQR)
Gestational age at surgery (in weeks)		39 (± 6.5)
Weight at surgery (in kg)	2.87 ± 0.98	
Gestational age at birth (in weeks)	31.94 ± 3.55	
Weight at birth (in kg)	1.56 ± 0.75	

	Range	Median
Surgery duration	25–52 min	34 ± 8 min
Anesthesia duration	52–67 min	59.5 min ± 4.3 min
Apnea events	None	
Hypotension	None	
Requirement for escalation of respiratory therapy postoperatively	None	

### 3.3 Adverse Events and Outcomes

There were no cardiopulmonary events observed during or after the procedure. None of the neonates needed escalation of respiratory support intraoperatively or NICU subsequently, including four BPD cases. None required inotropic support intraoperatively or in NICU. All the cases, which were admitted for surgery purposes, were discharged from the NICU after 2–4 h of observation (except cases 3 and 12). No additional surgeries were performed apart from the inguinal surgery. Further information about the cases can be found in Table 1.

## 4 Discussion

Neonatal anesthetic management is challenging due to physiological immaturity, increasing susceptibility to the cardiorespiratory effects of GA [9]. Preterm infants are especially vulnerable, with risks such as transitional circulation, comorbidities, and apnea. As a result, neonatal surgeries carry higher morbidity and mortality than in older children [10].

The choice between GA and SA often depends on institutional expertise. Interest in SA for high‐risk neonates began with Abajian et al. [11] in 1984, with subsequent studies highlighting its safety and reduced risk of apnea in preterm infants [12, 13, 14]. Veverka et al. [13] reported no apnea in 84 such cases, and Ali et al. [15] supported SA as a safe alternative for infraumbilical surgeries. Similarly, our cohort showed no perioperative apnea.

SA is safe and effective for lower abdominal and limb surgeries in extreme preterm infants with BPD, reducing perioperative cardiorespiratory risks and the need for prolonged monitoring. Compared to adults, infants exhibit fewer hemodynamic fluctuations, though rare hypotension warrants continuous intraoperative monitoring [16]. No hypotension occurred in our cohort. Despite its benefits, SA remains underutilized in India, likely due to institutional practices or limited expertise.

SA in neonates is well‐tolerated and safe, with no significant impact on cerebral oxygenation [8, 15, 17, 18]. SA is highly effective in term infants < 45 weeks postconceptional age (PCA) and expreterm up to 60 weeks PMA, with reported success rates up to 98% [19]. Although outcomes may vary by institutional expertise, our unit routinely uses SA for neonatal surgeries, achieving 100% success in this cohort.

SA in neonates has a shorter duration than in adults, making it suitable for procedures under 60 min [19]. In our center, all surgeries were completed within this timeframe without the need for supplemental anesthesia.

Our cohort had a mean gestational age of 31.94 ± 3.55 weeks and birth weight of 1.56 ± 0.75 kg, lower than those reported in other studies [15, 17]. At surgery, the median gestational age was 39 weeks and mean weight 2.87 ± 0.98 kg, again lower than those in cohorts by Randriamizao [17] (40.5 weeks and 3.45 kg) and Ali et al. (4.04 kg) [15]. This highlights our experience with younger and smaller neonates. All 19 infants remained stable, with no need for respiratory or inotropic support. Those admitted for surgery were discharged from the NICU within 2–4 h.

This study highlights SA as a safe alternative to GA for inguinal surgeries in infants, including those with BPD. This retrospective single‐center study with a small sample size may limit generalizability. However, the focused cohort allowed for detailed case‐level analysis of perioperative events and outcomes, providing meaningful clinical insights. Larger, multicenter studies are needed to validate these findings across broader populations. Our cohort included only infants who underwent surgery under SA, and we acknowledge the lack of comparative data with GA as a limitation.

## 5 Conclusion

This research presents further evidence supporting the effectiveness of SA in reducing or eliminating the risk of postoperative apnea in high‐risk preterm infants, including those with BPD undergoing inguinal hernia repair, even in the Indian setting. The study also highlights the technical feasibility and success of SA in most infants who undergo inguinal hernia repair. Moreover, the findings demonstrate that the use of SA eliminates the need for routine postoperative hospital admission for apnea monitoring in a high‐risk outpatient population of preterm infants, especially with BPD.

## Author Contributions

Conceptualization: Karthik Nagesh, Ranganatha A. Devaranavadagi, and Jayashree S. Simha. Data curation: Pavitra Gangadharan Chandrasekaran, Netra S. Kannur, Ranganatha A. Devaranavadagi, Gayatri Sasikumar, and Suma Sriramanan. Methodology: Karthik Nagesh, Ranganatha A. Devaranavadagi, Gayatri Sasikumar, Suma Sriramanan, H. A. Venkatesh, C. N. Radhakrishnan, and Jayashree S. Simha. Data analysis: Karthik Nagesh, Ranganatha A. Devaranavadagi, Gayatri Sasikumar, and Netra S. Kannur. Project administration and supervision: Karthik Nagesh, Jayashree S. Simha, C. N. Radhakrishnan, and H. A. Venkatesh. Writing – original draft: Karthik Nagesh and Ranganatha A. Devaranavadagi. Writing – reviewing and editing: Karthik Nagesh, Ranganatha A. Devaranavadagi, and Suma Sriramanan. All authors contributed to the article and approved the submitted version.

## Disclosure

The authors have nothing to report.

## Ethics Statement

The Ethics committee of Manipal Hospital, Bangalore, India, has issued no objection to publish the data—EC/RENEW/INST/2024/17014.

## Conflicts of Interest

The authors declare no conflicts of interest.

## Supporting information

Supplementary Material

## Data Availability

All data relevant to the study are included in the article. A supplementary file has been provided.
