# Supplementary material for: Early Forays Into Using Spinal Anesthesia for Inguinal Hernia Surgery in Preterm Infants: A Retrospective Analysis From a Single Center in Karnataka
Source: Pediatr Discov. 2025 Jul 24;3(3):e70015. doi: 10.1002/pdi3.70015 (PMC12483297; doi:10.1002/pdi3.70015)
Supplement: Supplementary file 1 — Supplementary Material [file PDI3-3-e70015-s001.docx]

|  | **Case1** | **Case2** | **Case3** | **Case4** | **Case5** | **Case6** | **Case7** | **Case8** | **Case9** | **Case 10** | **Case 11** | **Case 12** | **Case-13** | **Case 14** | **Case 15** | **Case 16** | **Case 17** | **Case 18** | **Case 19** |
| --- | --- | --- | --- | --- | --- | --- | --- | --- | --- | --- | --- | --- | --- | --- | --- | --- | --- | --- | --- |
| **Sex** | **Male** | **Male** | **Female** | **Male** | **Female** | **Female** | **Female** | **Male** | **Female** | **Male** | **Male** | **Female** | **Male** | **Female** | **Female** | **Female** | **Male** | **Male** | **Female** |
| **Birth weight (in kg)** | 1.35 | 1.095 | 0.85 | 3.3 | 0.75 | 1.3 | 2.06 | 1.42 | 0.99 | 1.43 | 0.96 | 0.74 | 1.9 | 1.69 | 2.6 | 0.77 | 2.4 | 2.72 | 1.29 |
| **Birth gestational age (in weeks)** | 31 | 29 | 28 | 38 | 27 | 32 | 32 | 29 | 28 | 33 | 32 | 27 | 33 | 35 | 38 | 31 | 36 | 37 | 31 |
| **Gestational age at surgery (in weeks)** | 42 | 37 | 37 | 46 | 38 | 42 | 39 | 37 | 37 | 39 | 37 | 35 | 37 | 44 | 44 | 43 | 42 | 42 | 39 |
| **Weight at surgery (in kg)** | 2.44 | 2.28 | 1.55 | 4.9 | 2.6 | 3.4 | 2.67 | 3.1 | 1.7 | 2.7 | 1.9 | 1.8 | 2.36 | 3.39 | 3.56 | 2.5 | 3.8 | 3.9 | 2.2 |
| **Surgery performed** | LIH | BIH | LIH | BIH | RIH | RIH | LIH | LIH | RIH | LIH | LIH | LIH | RIH | BIH | RIH | RIH | RIH | RIH | LIH |
| **Volume of spinal bupivacaine** | 0.5ml | 0.4ml | 0.3ml | 1ml | 0.5ml | 0.7ml | 0.5ml | 0.6ml | 0.3ml | 0.5ml | 0.4ml | 0.4ml | 0.5ml | 0.7ml | 0.7ml | 0.5ml | 0.8ml | 0.8ml | 0.5ml |
| **Level of spinal** | L4-L5 | L3-L4 | L4-L5 | L5-S1 | L3-L4 | L5-S1 | L3-L4 | L4-L5 | L5-S1 | L5-S1 | L3-L4 | L4-L5 | L4=L4 | L3-L4 | L4-L5 | L4-L5 | L3-L4 | L3-L4 | L5-S1 |
| **Hypotension during surgery** | No | No | No | No | No | No | No | No | No | No | No | No | No | No | No | No | No | No | No |
| **BPD** | No | No | Yes | No | Yes | No | No | No | Yes | No | No | Yes | No | No | No | No | No | No | No |
| **Requirement for escalation of respiratory therapy postoperatively** | No | No | No | No | No | No | No | No | No | No | No | No | No | No | No | No | No | No | No |

**Table -S1, showing Clinical and Surgical Data of Enrolled Neonates**

**(BPD: Bronchopulmonary dysplasia, IH: Inguinal hernia, RIH: Right Inguinal hernia, LIH: Left Inguinal hernia, BIH: Bilateral Inguinal hernia)**
